# Whole-Genome Sequencing of *Brevundimonas diminuta* XGC1, Isolated from a Tuberculosis Patient in Gujarat, India

**DOI:** 10.1128/genomeA.00686-15

**Published:** 2015-06-25

**Authors:** Arpita Ghosh, Khyati Chandratre, Abhinav Chaudhary, Spandan Chaudhary, Namrata Badani, Pooja S. Chaudhary, Dipali Dhawan, Srinivas Vudathala, Surendra K. Chikara

**Affiliations:** Xcelris Labs Ltd., Ahmedabad, India

## Abstract

We report the draft genome of *Brevundimonas diminuta* strain XGC1, isolated from a tuberculosis-infected patient in Gujarat, India. This study also reveals that the *B. diminuta* XGC1 strain has acquired mutation to confer resistance to quinolone drugs.

## GENOME ANNOUNCEMENT

*Brevundimonas* sp., previously assigned to the genus *Pseudomonas*, is a non-lactose-fermenting, aerobic, Gram-negative motile bacilli with a polar flagellum. *Brevundimonas* human infections are generally caused by *Brevundimonas vesicularis*, and only a few cases of severe opportunistic infections, particularly in patients with immunocompromised diseases, have been reported to be caused by *Brevundimonas diminuta*. *Brevundimonas* drug resistance has been reported in patients having cancer, and the infections were found in the bloodstream, intravascular catheter, urinary tract, and pleural space. The mechanism underlying drug resistance may involve GyrA, GyrB, ParC, and other genes (1).

The genome of *B. diminuta* strain XGC1 was sequenced using Illumina NextSeq at Xcelris Labs Ltd., Ahmedabad, India, with sequencing depth coverage of ~400×. *De novo* genome assembly was carried out using CLC Genomics Workbench version 6.0, which generated 31 scaffolds and a 3.1-Mb genome with 51% GC content.

The genome was annotated using the RAST (Rapid Annotations using Subsystem Technology) server (2). A total of 3,047 potential coding sequences (CDSs) were identified, which included 815 hypothetical genes and 48 RNA genes. Out of 48 RNA genes, 43 coded for tRNA and 5 for rRNA, which includes 3 large subunit, 1 small subunit of ribosome, and 1 5S RNA.

The GyrA QRDR region of *B. diminuta* strain XGC1 was compared with *P. aeruginosa, Campylobacter jejuni, B. diminuta* ATCC 11568 and *B. diminuta* 2192 for drug resistance*.* It is reported that amino acid at position 83 (Ser/Thr-83) and 87 (Asp/Glu-87) of the QRDR region of the GyrA subunit is responsible for drug resistance. The *B. diminuta* XGC1 strain was found to have Ala-83 and Met-87 mutation in the GyrA QRDR region, which confirms quinolone resistance.

### Nucleotide sequence accession numbers.

This whole-genome shotgun project has been deposited at DDBJ/EMBL/GenBank under the accession number LBNT00000000. The version described in this paper is the first version, LBNT01000000.
